# Vaccination status and needs of asylum-seeking children in Denmark: a retrospective data analysis

**DOI:** 10.1016/j.puhe.2018.02.018

**Published:** 2018-05

**Authors:** C.S. Nakken, M. Skovdal, L.B. Nellums, J.S. Friedland, S. Hargreaves, M. Norredam

**Affiliations:** a Danish Research Center for Migration, Ethnicity and Health, Section for Health Services Research, Department of Public Health, University of Copenhagen, Øster Farimagsgade 5, 1353, Copenhagen K, Denmark; b Infectious Diseases and Immunity, Department of Medicine, Imperial College London, Hammersmith Hospital, Du Cane Road, W12 0NN, London, UK; c Section of Immigrant Medicine, Department of Infectious Diseases, Copenhagen University Hospital, Hvidovre Hospital, Hvidovre, Denmark

**Keywords:** Asylum-seeking children, Vaccination, Immunisation, Denmark

## Abstract

**Objectives:**

Asylum seekers to Europe may come from war-torn countries where health systems have broken down, and there is evidence that asylum-seeking children have low coverage of childhood vaccinations, as well as uptake of immunisations in host countries. Such gaps in immunisation have important implications for effective national vaccination programmes. How we approach vaccination in children and adults entering Western Europe, where as a group they face barriers to health services and screening, is a growing debate; however, there are limited data on the vaccination status of these hard-to-reach communities, and robust evidence is needed to inform immunisation strategies. The aim of this study was to explore the vaccination status and needs of asylum-seeking children and adolescents in Denmark.

**Study design:**

We conducted a retrospective data analysis of anonymised patient records for asylum-seeking children and adolescents extracted from the Danish Red Cross database.

**Methods:**

We retrospectively searched the Danish Red Cross database for children and adolescents (aged 3 months–17 years) with active asylum applications in Denmark as of October 28, 2015. Data were extracted for demographic characteristics, vaccination status and vaccinations needed by asylum-seeking children presenting to Red Cross asylum centres for routine statutory health screening.

**Results:**

We explored the vaccination status and needs of 2126 asylum-seeking children and adolescents. About 64% of the study population were male and 36% were female. Eight nationalities were represented, where 33% of the total of children and adolescents were not immunised in accordance with Danish national guidelines, while 7% were considered partly vaccinated, and 60% were considered adequately vaccinated. Afghan (57% not vaccinated/unknown) and Eritrean (54% not vaccinated/unknown) children were the least likely to be vaccinated of all nationalities represented, as were boys (37% not vaccinated/unknown) compared with girls (27% not vaccinated/unknown) and children and adolescents aged between 12 and 17 years (48% not vaccinated/unknown) compared with 6- to 11-year olds (26%) and 0- to 5-year olds (22%). The health screenings resulted in 1328 vaccinations. The most commonly needed vaccines were diphtheria, tetanus, pertussis, polio and *Haemophilus influenzae* type b, (DTaP/IPV/Hib) which comprised 49% of the vaccines distributed, followed by the pneumococcal vaccine (Prevnar) (28%) and measles, mumps and rubella (MMR) vaccine (23%).

**Conclusions:**

The finding that nearly one-third of asylum-seeking children and adolescents in Denmark were in need of further vaccinations highlights the gaps in immunisation coverage in these populations. These results point to the need to improve access to health services and promote national vaccine programmes targeted at these communities to facilitate vaccination uptake and increase immunisation coverage to reduce the risk of preventable infectious diseases among asylum-seeking children.

## Highlights

• Little is known on the state of immunisation of refugees arriving in Western receiving countries.
• Variations in status on arrival to Denmark were associated with gender, country of origin and age groups.
• Vaccination needs were found to be associated with country of origin, while gender did not act as a significant determinant.
• Immunisations for vaccine-preventable diseases is critical for protecting refugee health and host communities.

## Introduction

A record number of 1.2 million first-time asylum seekers were registered in Europe in 2015;^1^ of whom, 21,225 applied for asylum in Denmark, an increase of 43% between 2014 and 2015. Various studies have shown that migrant populations present different patterns of infectious diseases and health-related behaviours than local-born populations.^2^, ^3^ Simultaneously, the limited immunisation programmes in countries of origin for asylum seekers in Europe (e.g. due to conflict or insufficient health service infrastructure^4^) have led to outbreaks of vaccine-preventable diseases in host countries in Europe, especially among young children.^5^ Poor living conditions before, during and after migration, barriers to accessing timely and appropriate health care (including for vaccination and screening for infectious diseases in host countries^6^) and limited health infrastructure in many countries of origin (particularly in the context of conflict) meant that very few of the children included in this study had documentation of their vaccinations on arrival. As a result, in many cases healthcare personnel had to rely on self-reported vaccination status from the children's families, which in many cases also required interpreters for translation. As a result, very limited information was ultimately available on children's vaccination status and needs.

To be granted asylum in Denmark, both adult and child asylum seekers must meet certain conditions based on the United Nations Refugee Convention or the conditions for protection status defined in section 7 of the Danish Aliens Act.^7^, ^8^, ^9^ All asylum seekers file their application in a reception centre run by the Danish Red Cross where they stay for the first weeks on arrival.^8^ The Danish health care system is built on the principle of equal access for all and universal health insurance. However, asylum seekers are only partly covered by this system. Child asylum seekers have the right to receive acute medical care. Furthermore, general practitioners and child health nurses are accessible at the asylum centres. In case of chronic disease or elective treatment, doctors at the asylum centres have to apply to the Danish Immigration Service to get costs covered.

Forty-five asylum centres existed in Denmark in 2017, which were run by the Danish Red Cross, the municipalities or both in a partnership. These include a reception centre, two departure centres, eight children centres and one ‘special care’ centre. All asylum seekers are on arrival accommodated in a reception asylum centre and are later referred to other asylum centres.^10^ Migrant populations are thought to have higher rates of vaccine-preventable diseases^6^ and potentially lower rates of vaccination coverage,^11^ with very low rates documented in children,^11^ yet data are lacking on vaccination status among migrants—and refugee and asylum-seeking children in particular—in Western Europe with which to inform policy in this area.^11^, ^12^ In Denmark, the government offers a statutory health check within 2 weeks of arrival to all migrants who make a claim for asylum, where additional immunisation screenings and vaccines are offered to all children and adolescents younger than 18 years old. Current vaccination protocols from the National Board of Health^13^ include administration of what is known as the ‘5-in-1 vaccine’, which covers diphtheria, tetanus, pertussis, polio and *Haemophilus influenzae* type b (DTaP/IPV/Hib) and the conjugated pneumococcal vaccine. The vaccine against measles, mumps and rubella (MMR) is also distributed, and at the age of 12 years, an additional MMR vaccination is offered if the child has not previously received both MMR vaccines. Finally, the two human papillomavirus (HPV) vaccinations are offered to girls at the age of 12 years.^13^, ^14^ Additionally, asylum-seeking children younger than 6 years are entitled to and offered vaccinations against hepatitis B.^13^ An overview of the Danish vaccination programme is given in Table 1. To address significant gaps in data on immunisations for refugee and asylum-seeking children in Europe, we aimed to explore the vaccination status and needs of all asylum-seeking children and adolescents with active asylum applications, presenting to statutory health checks at the Red Cross asylum centres in Denmark through a retrospective database analysis.

**Table 1:** Overview of the Danish vaccination programme: vaccines and age of distribution.

3 months	5 months	12 months	15 months	4 years	5 years	Younger than 6 years	12 years
DTaP/IPV/Hib	DTaP/IPV/Hib	DTaP/IPV/Hib	MMR	MMR	DTaP-IPV	Hepatitis B	MMR
PCV	PCV	PCV					HPV

DTaP/IPV/Hib, diphtheria, tetanus, pertussis, polio and *Haemophilus influenzae* type b; PCV, pneumococcal vaccine; MMR, measles, mumps and rubella; HPV, human papillomavirus.

## Methods

In this retrospective data analysis, we analysed anonymised patient records extracted from the Danish Red Cross database for asylum-seeking children and adolescents aged 3 months–17 years with active asylum applications in Denmark as of October 28, 2015 who presented to Red Cross centres for routine statutory health screening. Children may remain ‘active’ for long periods of time in the Red Cross database, depending on the amount of time it takes to process their applications or appeals. Consequently, the study did not necessarily reflect the most recent asylum-seeking population but rather all active children as of October 28, 2015 who were awaiting responses to their applications from the Danish Immigration Service. Data were extracted on demographic characteristics (e.g. age, gender, country of origin) and vaccination status. Vaccination need data were collected by accessing medical records in the Red Cross database that documented which vaccinations children had received while residing at Red Cross asylum centres in Denmark.

Vaccination needs were defined based on the healthcare personnel's assessments of which vaccines the asylum-seeking children were lacking on arrival and while having active asylum applications in Denmark, based on the Danish National Board of Health's current vaccination guidelines.^13^ Vaccinations were normally given at the reception centre within a fortnight of arrival.

Country groups with >100 children and adolescents were included for analyses. Furthermore, we categorised data into male/female and the following age groups based on being young children (0–5 years old), young school age (6–11 years old) and adolescents (12–17 years old), enabling us to compare young children with older children and adolescents. In the age group 0- to 5-year olds, it was decided that all children found to be younger than 3 months old would not be included because of limited data regarding their vaccination status on arrival, as they would normally be referred to follow the Danish vaccination programme from the beginning at 3 months old, regardless of prior status. During data processing, it was discovered that some children in the study population had turned 17 years old and hence were no longer within the age inclusion criteria, which led to their exclusion. The hepatitis B and HPV vaccination data were not included in the study because of inconsistencies and gaps in the data available. Descriptive analyses (e.g. percentages) were carried out for demographic characteristics. Multivariate logistic regression was conducted to examine the relationship between country of origin, age group, gender and vaccination status. Odds ratios and 95% confidence intervals were calculated and *P-*values below 0.05 were considered significant. Predictive value was measured using R-squared. All analyses were conducted in SPSS (version 22).

### Ethics

The National Board of Health and the Danish Data Protection Agency granted permission to use the data. Names, places, ID numbers or additional identifiers associated with the study population were anonymised before analysis.

## Results

A total of 2126 children had active asylum applications in the Danish Red Cross database as of October 28, 2015, 64% (1360) of whom were boys and 36% (776) of whom were girls. In all age groups, the male population dominated, with the highest number in the 12- to 17-year age group, 76% (640) of whom were boys, compared with 56% (408) in the 0- to 5-year age group and 54.3% (302) in the 6- to 11-year age group. In total, the largest number (39% [842]) of asylum-seeking children were between the ages of 12–17 years, followed by 34% (728) of children between the ages of 0–5 years and 26% (556) children in the group aged 6–11 years (Table 2).

**Table 2:** Age and gender distribution among asylum-seeking children in numbers and percentages.

Age	Female	Male	Total
0–5 years old	320 (44%)	408 (56%)	728 (100%)
6–11 years old	254 (45.7%)	302 (54.3%)	556 (100%)
12–17 years old	202 (24%)	640 (76%)	842 (100%)
**Total**	**776 (36%)**	**1350 (64%)**	**2126 (100%)**

As seen in Table 3, Syrians dominated the study population with 1014 children, constituting almost half of the total population (47.7%). The high number of Syrian children observed in the study population is likely to be attributed to the current situation in Syria. When considering the age distribution of each country, very similar numbers of children arrived from Syria in all age groups, with a minor increase in the group of 12- to 17-year olds (38%, 386). The situation was quite different for Afghans, where 67% (146) were between the ages of 12–17 years, compared with just 22% (23) of Somali children and 18% (34) of Russian children.

**Table 3:** Country and age distribution among asylum-seeking children in numbers and percentages.

Country	Age in years						Total	
	0–5		6–11		12–17			
Syria	335	33%	293	29%	386	38%	1014	100%
Afghanistan	43	20%	28	13%	146	67%	217	100%
Russia	85	46%	66	36%	34	18%	185	100%
Stateless Palestinians	56	30%	66	36%	62	34%	184	100%
Iran	60	36%	52	31%	56	33%	168	100%
Iraq	48	36%	31	23%	55	41%	134	100%
Eritrea	26	22%	13	11%	80	67%	119	100%
Somalia	75	71%	7	7%	23	22%	105	100%
**Total**	**728**	**34%**	**556**	**26%**	**842**	**40%**	**2126**	**100%**

### Vaccination status

**Fig. 1:**
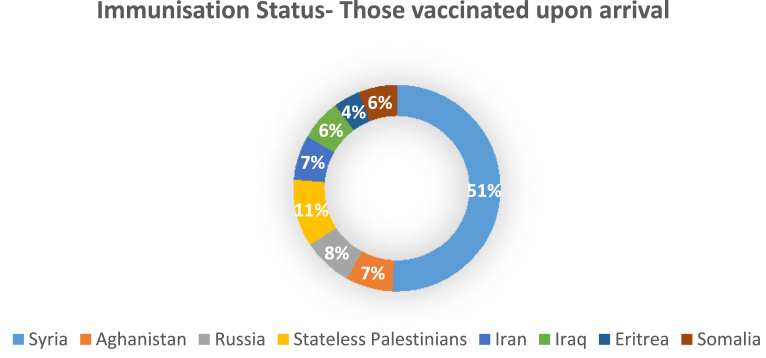
Immunisation status on arrival among asylum-seeking children (*n* = 2126).

About 33% of asylum-seeking children and adolescents did not have adequate vaccinations in line with Danish national guidelines, while 7% were considered partly vaccinated. Thirty-seven percent of boys were found to not be vaccinated or to have unknown vaccination status as compared with 27% of girls. The least likely children to be vaccinated were from Afghanistan (57% not vaccinated or with unknown vaccination status) and Eritrea (54% not vaccinated or with unknown vaccination status). The distribution by country of origin of children who were adequately vaccinated on arrival in Denmark is presented in Fig. 1.

As displayed in the multivariate regression analysis in Table 4, those found to have the lowest odds of being vaccinated were the children from the countries of Afghanistan (odds ratio [OR] = 0.50, 95% confidence interval [CI] 0.37–0.67) and Eritrea (OR = 0.53, 95% CI 0.36–0.79). Contrary to this, the country groups presenting the highest odds of being vaccinated were the Somalis (OR = 1.48, 95% CI 0.93–2.34) and stateless Palestinians (OR = 1.50, 95% CI 1.05–2.14). The odds of being vaccinated were also higher among children between the ages of 0–5 years (OR = 1.58, 95% CI 1.27–1.95) and 6–11 years (OR = 1.79, 95% CI 1.41–2.26), compared with children aged 12–17 years. Girls were found to have a lower odds of being vaccinated compared with boys (OR = 0.80, 95% CI 0.66–0.97). Country of origin (*P* < 0.001), age (*P* < 0.001) and gender (*P* < 0.025) were all found to be significantly associated with vaccination status. In the model, the R-squared value was calculated to show how much of the variability is explained by the predictors. The model was able to predict 60.1% of the outcomes.

**Table 4:** Regression analysis of asylum-seeking children considered vaccinated according to country, age group and gender (n=2,126).

Characteristic	% vaccinated	No. vaccinated	OR (95% CI)	*P*-value
**Country of origin**				<**0.001**
Syria	52%	731	1	
Afghanistan	7%	102	0.50 (0.37–0.67)	
Eritrea	4%	55	0.53 (0.36–0.79)	
Iran	7%	99	0.61 (0.44–0.86)	
Iraq	6%	89	0.94 (0.65–1.37)	
Russia	8%	114	0.58 (0.42–0.81)	
Somalia	6%	80	1.48 (0.93–2.34)	
Stateless Palestinians	11%	150	1.50 (1.05–2.14)	
**Age group**				<**0.001**
12–17 years old	31%	441	1	
6–11 years old	29%	410	1.79 (1.41–2.26)	
0–5 years old	40%	569	1.58 (1.27–1.95)	
**Gender**				**0.025**
Male	60%	854	1	
Female	40%	566	0.80 (0.66–0.97)	

OR, odds ratio; CI, confidence interval

*P* ≤ 0.05.

### Vaccination needs

**Fig. 2:**
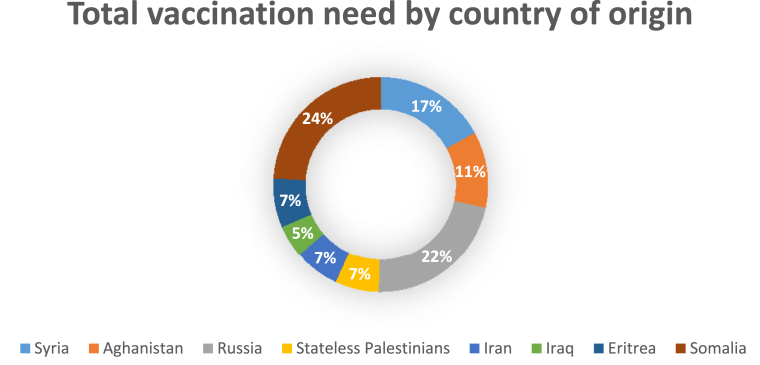
Overview of total vaccination needs by country of origin (n = 1,328).

As demonstrated in Table 5, a total of 1328 vaccinations were distributed to the 2126 children included in the study after arrival at the Red Cross asylum centres as of October 28, 2015. About 802 vaccines were distributed to boys, with DTaP/IPV/Hib being the most needed vaccination comprising 49% (395) of vaccines distributed, followed by the Prevnar with 28% (224) and MMR with 23% (183). A total of 526 vaccinations were distributed to girls, again with the DTaP/IPV/Hib vaccines being the ones most needed (48%, 251), followed by the Prevnar (31%, 165) and MMR (21%, 110). Country of origin was found to be associated with vaccination needs (*P* < 0.003). However, gender was not found to be significantly associated with vaccination needs (*P* = 0.376). A total of 114 DTaP/IPV/Hib vaccinations were distributed to Syrian children, who formed the largest country group in the study, while the highest number of DTaP/IPV/Hib vaccinations was needed by the Somalis (142 vaccinations). The Somalis were also the ones most in need of Prevnar vaccines, with 122 vaccines distributed, a high number compared with the stateless Palestinians who received 13. Russian and Syrian children had the highest need of MMR vaccinations, with 59 and 58 vaccinations given, respectively. The Iraqis (5%), stateless Palestinians (7%), Iranians (7%) and Eritreans (7%) received the lowest number of total vaccinations distributed (Fig. 2).

**Table 5:** Overview of vaccination needs by gender and country of origin (n=1,328).

Characteristic	Vaccination needs						Total number of vaccinations distributed		*P*-value
	DTaP/IPV/Hib		All Prevnar		All MMR				
	#	%	#	%	#	%	#	%	
**Gender**									0.376
Male	395	49%	224	28%	183	23%	802	100%	
Female	251	48%	165	31%	110	21%	526	100%	
**Country of origin**									0.003
Syria	114	51%	53	24%	58	26%	225	100%	
Afghanistan	78	51%	40	26%	34	22%	152	100%	
Russia	137	47%	94	32%	59	20%	290	100%	
Stateless Palestinians	48	55%	13	15%	27	31%	88	100%	
Iran	41	46%	23	26%	26	29%	90	100%	
Iraq	34	52%	17	26%	14	22%	65	100%	
Eritrea	51	52%	27	28%	20	20%	98	100%	
Somalia	142	45%	122	38%	55	17%	319	100%	

DTaP/IPV/Hib, diphtheria, tetanus, pertussis, polio and *Haemophilus influenzae* type b; MMR, measles, mumps and rubella.

## Discussion

The study found significant relationships between country of origin, age, gender and vaccination status on arrival in Denmark. Afghans and Eritreans presented significantly lower vaccination rates compared with Syrians and stateless Palestinians. Higher odds of being vaccinated were associated with being male, being a younger child and being a Somali, Syrian or a stateless Palestinian (previously residing in Syria). Vaccination needs in Denmark were found to be related to the country of origin of the asylum-seeking children, with the greatest number of vaccines being provided to children from Somalia and Russia, and the fewest vaccinations provided to children from Iraq and to stateless Palestinians. Gender did not act as a significant determinant.

The few studies that have explored immunisation concerns among asylum seekers and refugees in Western receiving countries have highlighted country of origin as an important determinant of immunisation coverage,^15^, ^16^ a finding which this study also affirmed. However, we also found that other contributing factors exist. We established that stateless Palestinians and Syrian children were assessed to be well vaccinated before arrival, which is in line with statements from the United Nations,^17^ affirming that many current refugees are coming from Middle Eastern countries where immunisation coverage has been historically high.^17^ Yet, important aspects to consider are setbacks in immunisation programmes due to conflict and also the time the children have been migrating.

Even with ongoing high vaccination coverage, the current situation, with high numbers of asylum seekers arriving in European countries, poses new challenges for healthcare workers regarding where and when to vaccinate. The situation is intensified by the fact that many vaccines must be given in repeated doses at specific intervals, which is difficult to ensure when people are constantly on the move.^17^ This may help to clarify why children from some countries were still assessed to have vaccination needs on arrival despite high vaccination coverage in their country of origin. Although a national immunisation programme exists, resources devoted to immunisation delivery are likely to vary by district and region in the different countries. Such socio-economic disparities may account for the variances found in the study population. Correspondingly, other studies have recognised socio-economic deprivation, rather than ethnicity, as the dominant determinant of immunisation coverage.^18^

Interestingly, our study identified not only high levels of immunisation coverage among children in Somalia but also found that asylum-seeking Somali children were most in need of vaccinations on arrival, needing the highest number of total vaccinations of all the country groups. These contrary findings indicate that there are factors related to the migration process, rather than solely factors related to the country of origin, that influence the children's state of immunisation. In this context, according to the World Health Organization (WHO) et al.,^17^ those most at risk are children who have not yet been vaccinated because the vaccination programmes in their countries have been interrupted by war and civil conflicts, which is likely to lead to increasing numbers of undervaccinated child and adolescent asylum seekers to Europe over time. Even though many arrive from countries with relatively high immunisation coverage, a large portion arrive from endemic areas and have travelled under conditions that increase the risk of acquiring vaccine-preventable diseases. Many also continue to be exposed to poor hygienic circumstances and overcrowding after arrival in Europe.^19^ Furthermore, our study encountered variability in immunisation status between age groups, revealing that the youngest age groups had the highest odds of being vaccinated before arrival and thus suggesting that it is the oldest children who are most in need of vaccinations on arrival. Yet, it has to be taken into account when interpreting the data that vaccination records may not be available for some children because of poor access to healthcare and vaccination records in countries of origin before migration, loss of records in transit, poor record-keeping and access to records during the migration trajectory or reluctance to provide such records in light of the Dublin agreement.^8^ Previous research has been divided on the relationship between age and immunisation status. Some studies have found that immunisation coverage is lower in older children,^18^, ^20^ while others have established that younger children are more likely to be unvaccinated because of premigration factors such as interruptions to vaccination programmes because of conflicts/wars.^17^

Our findings are constrained by the fact that our data are based on healthcare personnel's assessment of families' self-reported vaccination history. While this may explain some of the differences observed, the alternative (serological testing of each child) would be both costly and logistically complicated. Instead, many countries like Denmark provide revaccination or boosters according to national child vaccination programmes. This demonstrates that the assessed immunisation status and needs are highly influenced by factors related to national guidelines in receiving countries, as well as political and economic aspects within each country's healthcare system. Although there are significant variations in vaccination policies for migrants across European countries, there have been calls for evidence-based European guidance for the prevention of infectious diseases, including immunisations, in migrants to Europe, particularly in light of the recent refugee crisis,^20^ and recent WHO-UNHCR-UNICEF joint technical guidance^17^ stipulates migrants should be vaccinated according to the immunisation schedules of countries in which they intend to stay for more than a week.^17^ In light of the clear impetus to improve the provision of preventative care to these hard-to-reach populations, the approach to immunisations in migrants we report here in Denmark may serve as a model for other European countries for routine health checks and the provision of vaccinations to migrant children.

### Conclusion

Significant disparities in vaccination status and needs were found between different groups of asylum-seeking children and adolescents. This should lead to reflections on current immunisation assessments and a search for strategies to develop evidence-based preventive services to reduce the risk of vaccine-preventable infectious diseases in these communities and, consequently, poor and costly health outcomes. This should be supported by targeted initiatives to promote vaccination in groups with the greatest immunisation needs, as well as efforts to facilitate access to services for these hard-to-reach groups, particularly in light of increasingly restrictive healthcare policies across Europe.^21^ Further evidence is also needed around how healthcare personnel determine children's vaccination needs and effective (and cost-effective) approaches to identifying vaccination status to be able to implement adequate guidelines and global standards to assist with the immunisation of children on the move.

## Author statements

### Acknowledgements

The authors would like to thank the Danish Red Cross Asylum Department for their time and efforts. They appreciate the support and collaboration from the Danish Red Cross, especially chief medical consultant Ebbe Munk Andersen and codirector Svend Erik Brande.

### Ethical approval

The National Board of Health and the Danish Data Protection Agency granted permission to use the data. Names, places, ID numbers or additional identifiers associated with the study population were anonymised before analysis.

### Funding

L.B.N., S.H. and J.S.F. receive funding from the UK National Institute for Health Research Imperial Biomedical Research Centre, the Imperial College Healthcare Charity, the Wellcome Trust (Grant Number 209993/Z/17/Z) and European Society for Clinical Microbiology and Infectious Diseases (ESCMID) research funding through the ESCMID Study Group for Infections in Travellers and Migrants (ESGITM).

### Competing interests

None declared.
